# Scoping review of military veterans involved in the criminal legal system and their health and healthcare: 5-year update and map to the Veterans-Sequential Intercept Model

**DOI:** 10.1186/s40352-024-00274-9

**Published:** 2024-04-19

**Authors:** Kreeti Singh, Christine Timko, Mengfei Yu, Emmeline Taylor, Jessica Blue-Howells, Andrea K. Finlay

**Affiliations:** 1 Center for Innovation to Implementation, VA Palo Alto Health Care System, 795 Willow Road (152-MPD), Menlo Park, CA 94025 USA; 2 Department of Psychiatry and Behavioral Sciences, Stanford University School of Medicine, 291 Campus Drive, Li Ka Shing Building, Stanford, CA 94305 USA; 3 Department of Veterans Affairs, National Center on Homelessness Among Veterans, 795 Willow Road, Menlo Park, CA 94025 USA; 4 Department of Veterans Affairs, Veterans Justice Programs, 810 Vermont Avenue, Washington DC, NW 20420 USA; 5 Department of Psychology, University of Colorado, Columbine Hall 4th Floor, 1420 Austin Bluffs Pkwy, Colorado Springs, CO 80918 USA

**Keywords:** Military health, Veterans, Criminal justice, Healthcare, Jail, Prison, United States Department of Veterans Affairs, Sequential intercept model

## Abstract

**Background:**

A previous scoping review of legal-involved veterans’ health and healthcare (1947–2017) identified studies and their limitations. Given the influx of literature published recently, this study aimed to update the previous review and map articles to the Veterans-Sequential Intercept Model (V-SIM) – a conceptual model used by key partners, including Veterans Health Administration, veteran advocates, criminal justice practitioners, and local governments to identify intercept points in the criminal legal system where resources and programming can be provided. Developing an updated resource of literature is essential to inform current research, discover gaps, and highlight areas for future research.

**Methods:**

A systematic search of 5 databases identified articles related to legal-involved veterans’ health and healthcare published between December 2017 through December 2022. The first and senior authors conducted abstract reviews, full-text reviews, and data extraction of study characteristics. Finally, each article was sorted by the various intercept points from the V-SIM.

**Results:**

Of 903 potentially relevant articles, 107 peer-reviewed publications were included in this review, most related to mental health (66/107, 62%) and used an observational quantitative study design (95/107, 89%). Although most articles did not explicitly use the V-SIM to guide data collection, analyses, or interpretation, all could be mapped to this conceptual model. Half of the articles (54/107, 50%) collected data from intercept 5 (Community Corrections and Support Intercept) of the V-SIM. No articles gathered data from intercepts 0 (Community and Emergency Services Intercept), 1 (Law Enforcement Intercept), or 2 (Initial Detention and Court Hearings Intercept).

**Conclusions:**

There were 107 articles published in the last five years compared to 190 articles published in 70 years covered in the last review, illustrating the growing interest in legal-involved veterans. The V-SIM is widely used by front-line providers and clinical leadership, but not by researchers to guide their work. By clearly tying their research to the V-SIM, researchers could generate results to help guide policy and practice at specific intercept points. Despite the large number of publications, research on prevention and early intervention for legal-involved veterans is lacking, indicating areas of great need for future studies.

**Supplementary Information:**

The online version contains supplementary material available at 10.1186/s40352-024-00274-9.

Military veterans involved in the criminal legal system often have difficulty finding housing and employment and have elevated risks for suicide and overdose deaths (Finlay et al., 2022; McDonough, 2015; Palframan et al., 2020). Veterans are considered legal-involved if they have had contact with law enforcement, are involved in criminal court proceedings, are incarcerated in jails or prisons, or are under supervision by probation or parole (Finlay et al., 2019a, b, c). A prior scoping review focused on veterans in the criminal legal system and their health and healthcare identified 190 articles, with topic areas ranging from mental health conditions and treatment utilization (68% of studies), homelessness (13%, 24/191), access and utilization (7%, 14/191), psychosocial (5%, 10/191), and medical conditions (5%, 10/191). Ultimately, the review revealed gaps in the literature on legal-involved veterans for the following areas: (1) different sociodemographic groups, (2) the impact of managing multiple medical, mental health, and substance use disorders, (3) differences in health and healthcare by the type of criminal legal involvement, and (4) the lack of conceptual modeling and randomized trials. The current study aimed to update the prior scoping review and explore new developments in research on veterans in the criminal legal system.

## Veterans-Sequential Intercept Model

Although studies on legal-involved veterans often lack a conceptual model, the Veterans Justice Programs, the National Institute of Corrections, and the Justice Involved Veterans Network have developed the Veterans-Sequential Intercept Model (V-SIM) to align resources and support for veterans at distinct points in the criminal legal system (Veteran intercepts in the criminal justice system, 2023). The purpose of the V-SIM is to help engage a wider group of agencies who can deliver responsive services to address the unique needs of veterans. To contribute to the usefulness of this conceptual model in guiding work by clinical and criminal legal front-line providers and leadership, researchers need to utilize the V-SIM in their scientific studies. However, the extent to which researchers utilize the V-SIM to guide their research and recommendations focused on legal-involved veterans is unknown. This scoping review aims to map the extant literature to the V-SIM to determine gaps in existing literature at any of the intercept points in the criminal legal system as practitioners’ interface with veterans.

**Fig. 1:**
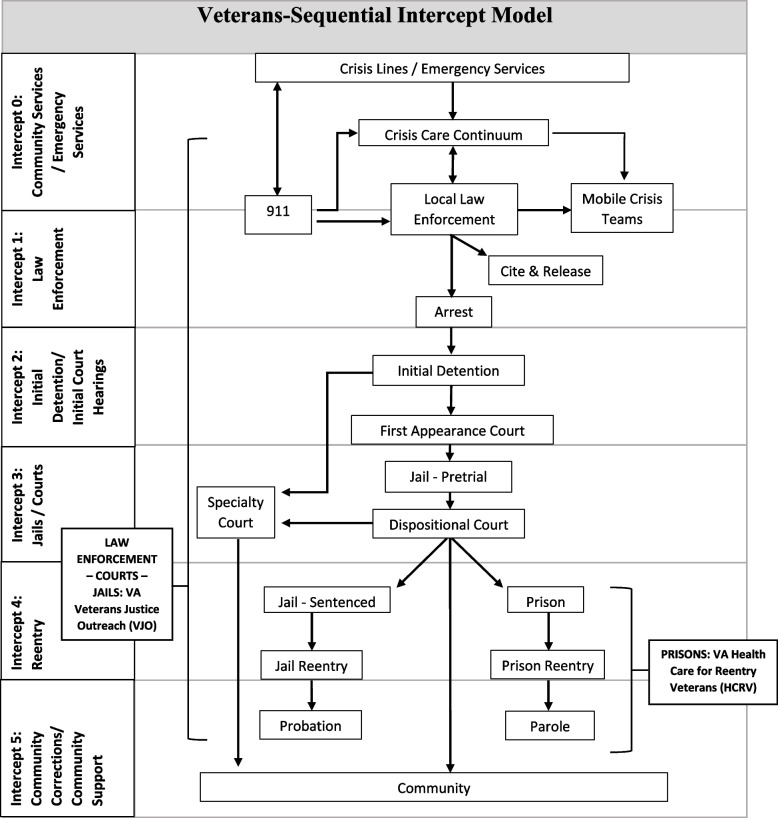
Veterans-Sequential Intercept Model (adapted from Munetz & Griffin, 2006 and Blue-Howells et al., 2013)

The V-SIM has six intercept points, adapted from Munetz and Griffin (2006) and Blue-Howells et al. (2013) (Fig. 1). The idea is to “intercept” individuals as early as possible to prevent them from progressing further into the criminal legal system and to provide treatment for those already in the system to reduce recidivism. Intercept 0, Community and Emergency Services Intercept, includes community services, such as crisis lines and crisis care continuum (e.g., walk-in urgent care, hospitalizations), and emergency services. Deflection and prearrest diversion programs in intercept 0 have the goal of preventing contact with the criminal legal system (Charlier & Reichert, 2020). Intercept 1, Law Enforcement Intercept, includes deflection to crisis teams, cite and release, and arrest. Intercept 2, Initial Detention and Court Hearing Intercept, includes initial detention and initial court hearings and follows individuals in the criminal legal system from arrest to their first appearance in court. Intercept 3, Jails and Courts Intercept, includes pre-trial jails and courts, such as dispositional courts or specialty courts, like Veterans Treatment Courts. Intercept 4, Reentry Intercept, is reentry into the community from jail or prison. We also added jail-sentenced and prison to intercept 4 for the purpose of categorizing the literature, because veteran-specific programming can be delivered in correctional facilities. Finally, intercept 5, Community Corrections and Community Support Intercept, is when veterans are released into the community on probation or parole or without any criminal legal supervision.

## Innovations for legal-involved veterans

Over the last 15 years, several innovative practices were developed to address treatment needs for legal-involved veterans. One innovation delivered by the Veterans Health Administration (VHA) is direct outreach by VHA to veterans at all points in the V-SIM. Outreach is conducted by VHA staff from the Veterans Justice Programs (VJP), which includes outreach to prisons through the Health Care for Re-entry Veterans (HCRV) program and outreach to jails, courts, and law enforcement through the Veterans Justice Outreach (VJO) program. The model of the program is to provide outreach to veterans at their point of involvement with the legal system (arrest [intercept 1], detention [intercept 2], jails/courts [intercept 3], jail/prison incarceration and reentry [intercept 4], and community corrections [intercept 5]), to provide an individualized assessment, and connect veterans with healthcare interventions that can assist in their recovery and minimize further contact with the legal system. Veterans Treatment Courts, part of intercept 3 (Jails and Courts Intercept), are one of the most widely disseminated innovative practices with more than 600 veteran-specific courts, dockets, and tracks throughout the United States (Finlay, 2019; Stewart, 2021). Veterans Treatment Courts are specialty courts that focus on connecting veterans to treatment, most commonly for mental health and substance use disorders, in lieu of incarceration (Douds & Hummer, 2019). The Veterans Treatment Court Improvement Act of 2018, Law Number 115–240, required the Department of Veterans Affairs (VA) to hire at least fifty VJO Specialists to provide treatment court services to legal-involved veterans and serve as liaisons between the courts and the VHA (“H.R.2147–115th Congress (2017–2018): Veterans Treatment Court Improvement Act of 2018,” 2018). With the introduction of this law, legal-involved veterans in the Jails and Courts Intercept (intercept 3), have received more guidance and support from the VJP.

More recent innovations have focused on helping veterans before a crisis or arrest occurs (intercept 0 [Community and Emergency Services Intercept] and intercept 1 [Law Enforcement Intercept]) or diverting them away from court or incarceration. These programs, called deflection and prearrest diversion, are collaborative and designed to treat people with mental health and substance use disorders to prevent entry into the legal system (Charlier & Reichert, 2020). They connect people to healthcare, housing, and other social services with the goal of avoiding arrest or further criminal legal contact. A recent study of veteran-specific cooperative police interventions found that veterans who received a police intervention had higher use of mental health and substance use disorder treatment, rehabilitation, and other services after 6 months, demonstrating the importance of collaborative relationships between local police departments, VA police, and the VJO Specialist (Tsai et al., 2023). These programs focus on intercept 0 (Community and Emergency Services Intercept) and intercept 1 (Law Enforcement Intercept).

## Current study

Since the 2017 review, there has been an increase of research on legal-involved veterans. The original scoping review was the first to examine the health and healthcare of legal-involved veterans and has been cited over 50 times since its 2019 publication (Google Scholar). Given the influx of research and growing interest in the topic of legal-involved veterans over the past five years, this study has two objectives: (1) provide an updated comprehensive list of existing literature on legal-involved veterans’ health and healthcare, and (2) map publications to the V-SIM to evaluate the connection between research and practice. Our study is designed to explore new developments that have occurred in the last five years and to inform policy and practice, identify research gaps that remain unaddressed, and guide future research directives specific to military veterans in the criminal legal system using the V-SIM.

## Methods

This scoping review presents an overview of all articles focused on legal-involved veterans and their health and healthcare but does not evaluate or present study findings. Ethical approval was not required because this study included only published manuscripts and reports.

### Data sources and searches

The original scoping review in 2017 followed a modified version of the PRISMA guidelines (Liberati et al., 2009) and scoping review guidelines (Peters, 2015). Following this same procedure (Finlay et al., 2019a, b, c), we conducted a systematic search of five databases: MEDLINE/PubMed, Scopus, Web of Science, CINAHL, and PsychINFO. We restricted the date range for the search from December 1, 2017, where the prior review ended, to December 31, 2022. Studies were eligible for inclusion if they appeared in our database search. Key terms, such as *veteran* and *former military*, and other criminal legal-related terms, including *prison, jail*, and *court* were used in each search engine (see Additional file 1 for search algorithms and terms used). Studies were limited to English language publications.

### Study selection

After compiling all articles identified in the search engines, duplicates were removed. Articles were uploaded to Rayyan (Ouzzani et al., 2016) for abstract review. Each abstract was reviewed independently by two reviewers (KS and AKF). Articles excluded at this stage were not related to health or healthcare or did not focus on legal-involved veterans. Articles without abstracts were included in this initial stage. Any discrepancies between reviewers were discussed and resolved.

After the abstract review, pdfs were collected for the remaining references to conduct full-text review. The first author was the primary reviewer for ~ 70% of articles and read all the articles. The last author also read all the articles and provided checks for the first author’s reviews. Any uncertainty about retaining an article at the full-text review stage was discussed between the two primary reviewers until consensus was reached. Consistent with previous studies, the following article types were excluded: editorials, magazine articles, case reports, conference abstracts, protocols, narrative/systematic reviews, unpublished work, vignette studies, and books/book chapters (Danan et al., 2017; Finlay et al., 2019a, b, c). We excluded any articles that were not peer-reviewed (Hartling et al., 2017). Also excluded were articles that did not include results specific to legal-involved veterans, were not relevant to health or healthcare, or focused on a population other than legal-involved veterans (e.g., active-duty military personnel). Finally, we excluded articles that were included in the 2017 scoping review, that is, manuscripts that were available as advanced online publications in 2017 and subsequently published.

### Data extraction

For articles included at the full-text review stage, we extracted the same study characteristics as the prior scoping review (Finlay et al., 2019a, b, c), to keep results comparable. The characteristics were: (1) healthcare category, (2) subcategory, (3) study design, (4) sample size, (5) percentage of veterans in the sample, (6) percentage of legal-involved veterans in the sample, (7–9) reporting of sex, race, and age, (10) research setting, (11) period of military service, (12) population, (13) outcomes reported, (14) funding source, (15) country, and (16) period of data collection (Appendix 2). Author names and article year of publication were also recorded. We did not calculate inter-rater reliability as any disagreements were discussed and resolved.

Articles were mapped to the adapted V-SIM (Fig. 1) by the intercept point that: (1) data were collected from, (2) was of interest at analysis, and (3) pertained to the recommendation for intercept intervention. “Intercept point data were collected from” refers to the study sample. For example, if the sample was post-criminal legal involvement, the intercept point that data were collected from was intercept 5 (Community Corrections and Support Intercept). “Intercept of interest at analysis” is the intercept point the study explicitly examined. For example, a study that used data from veterans incarcerated in prison (intercept 4, Reentry Intercept) but was examining a question about what occurred during the veteran’s arrest (intercept 1, Law Enforcement Intercept) would be coded as intercept 1 for the intercept of interest at analysis. Finally, “recommendation for intercept intervention” refers to the intercept point that conclusions were made.

In addition to intercepts 0 through 5, we added “general,” “not applicable,” and “PreSIM” as options to select while mapping to the intercept model. “General” was chosen for articles that gathered or analyzed data or made recommendations more broadly rather than focusing on any point of criminal legal involvement. Articles focusing on veterans leaving military service and prior to their legal involvement were categorized as “PreSIM”. Finally, “not applicable” was used primarily for mapping to the intercept point where data is collected from. There were studies that sampled staff members rather than legal-involved veterans, and some that sampled a combination of legal-involved veterans and staff members, or legal-involved veterans compared to non-legal-involved veterans. These articles did not include legal-involved veterans in the study sample exclusively, but we included them because they are directly relevant to the resources and support services offered to legal-involved veterans.

### Data synthesis and analysis

As each article was read, the sixteen study characteristics were extracted, and articles were mapped to the V-SIM. Next, the studies were summarized across characteristics and V-SIM intercept points. As a final check, we randomly selected ten articles to ensure our data extraction remained consistent throughout the coding process. Because our coding changed for three of the ten articles, both reviewers went through all 107 articles to double check the data that were extracted. Our results present an overview of the literature and do not examine the individual findings of each article. Risk of bias and other study details were not assessed for each article as scoping reviews are designed to map literature, not make analytical comparisons (Peters et al., 2022).

## Results

### Scoping review update

**Fig. 2:**
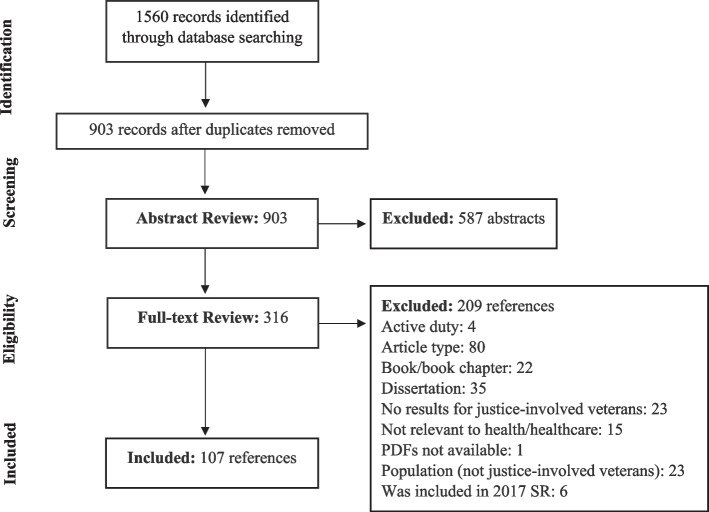
Adapted flow chart of record identification and screening process

There were 1,560 articles identified across five search engines (Fig. 2). After removing duplicates, 903 articles remained. Of these, 587 abstracts were excluded, leaving 316 articles for full-text review. There were 209 articles excluded at the full-text review stage, primarily because of article type (80 articles excluded). The final scoping review included 107 articles, all listed in Table 1, summarized by author, year, healthcare category, subcategory, and intercept points of data collection, analysis, and recommendations. Most articles focused on the mental health of veterans in the criminal legal system (66/107, 62%) followed by studies on these veterans’ access to and utilization of healthcare (10/107, 9%). Most research studies were conducted in the United States (98/107, 92%). However, some studies examined veterans in other countries, including the United Kingdom (5%, 5/107), Sweden (1%, 1/107), and Canada (3%, 3/107).

**Table 1:** Complete list of included articles and V-SIM mapping

**Author (Year)**	**Healthcare category**	**Subcategory**	**Intercept point data is collected from**	**Intercept of interest at analysis**	**Recommendation for intercept intervention**
Finlay et al. (2020)	Access and Utilization	Barriers and Facilitators of Care	5 and N/A	Gen	Gen
Kim et al. (2019b)			N/A	4 and 5	4
Morse et al. (2021)			5	Gen	4 and 5
Simmons et al. (2022)			5 and N/A	4 and 5	4
Taylor et al. (2022)			N/A	1 through 5	Gen
Timko et al. (2020b)			N/A	Gen	Gen
Timko et al. (2020c)			5	Gen	Gen
Tsai et al. (2018)			5	5	Gen
Finlay et al. (2021)		Healthcare Utilization	5	Gen	Gen
Harris et al. (2021)			5	Gen	Gen
Davis et al. (2020)	Healthcare Organization and Delivery	Electronic Health Record	5	Gen	Gen
Wang et al. (2019)			5	Gen	Gen
Blonigen et al. (2021)		Mental Healthcare Programming	N/A	5	5
Coté et al. (2020)			4	Gen	4
Rodriguez et al. (2019)			N/A	2 through 5	3 and 5
Sylvia et al. (2021)			4	4	4
Harris et al. (2018)	Homelessness	Multiple Mental Health, Substance Use Disorder and/or Medical Conditions	5	Gen	Gen
Kertesz et al. (2021)			5	Gen	Gen
Orak et al. (2022)			5	1	PreSIM
Szymkowiak et al. (2022)			5	5	4
Byrne et al. (2022)		Other Mental Health Topics	5	5	5
LePage et al. (2021b)		Vocational Training	5	5	5
Drapela et al. (2019)	Medical	Brain Injury	4	4	4
Hawks et al. (2022)		Death	5	4	4
Hawks et al. (2020)		Other Medical Topics	5	4	4 and 5
Khan et al. (2019)			5	4	4 and 5
Kuffel et al. (2022)			PreSIM, 4, and 5	4	0 and 1
McCall and Tsai (2018a)			4	4	4 and 5
Ottomanelli et al. (2022)			5	Gen	Gen
Blonigen et al. (2022)	Mental health	Mental Health Programming	5 and N/A	Gen	5
Canada et al. (2020)			5	1	Gen
Crowe et al. (2020)			3	3	3
Edwards et al. (2022a)			5	5	5
Goggin et al. (2018)			4	4	4
Holliday et al. (2022a)			5 and N/A	Gen	Gen
Kim et al. (2019a)			5 and N/A	4 and 5	4 and 5
Morgan et al. (2019)			4	4	3 through 5
Short et al. (2018)			5	0 and 1	0 and 1
Barr et al. (2022)		Multiple Mental Health, Substance Use Disorder and/or Medical Conditions	5	1	PreSIM
Barry et al. (2018)			5	4 and 5	4 and 5
Bhalla et al. (2020)			5	5	Gen
Chintakrindi and Gupta (2021)			4	4	Gen
Comartin et al. (2022)			4	4	4 and 5
Edwards et al. (2021)			5	Gen	Gen
Edwards et al. (2022)			5	1	PreSIM
Edwards et al. (2022b)			4	4	4
Edwards et al. (2022)			5	1	Gen
Elbogen et al. (2018)			5	Gen	Gen
MacDonald et al. (2022)			4	4 and 5	4 and 5
Finlay et al. (2018b)			5	Gen	Gen
Fletcher et al. (2022)			5	Gen	Gen
Holliday et al. (2021e)			5 and N/A	5	5
Logan et al. (2021a)			4 and 5	1 through 5	4
MacDonald et al. (2022)			4 and 5	2 through 5	Gen
Olusanya (2021)			3	3	3
Ross et al. (2018)			5	PreSIM	PreSIM
Stefanovics et al. (2022)			5	Gen	Gen
Stimmel et al. (2018)			5	Gen	Gen
Tsai et al. (2022)			5	4	5
Browne and Mohamed (2021)		Other Mental Health Topics	5	Gen	Gen
Mok et al. (2018)			N/A	1	1
Schaffer and Fulmer (2018)			4 and 5	1 through 5	5
Weaver et al. (2022)			N/A	1	1
Camins et al. (2022)		PTSD and/or Trauma	5	0 and 1	0
Logan et al. (2021b)			4 and 5	4 and 5	4 and 5
MacManus et al. (2021)			PreSIM through 5	Gen	Gen
Miles et al. (2021)			PreSIM and 5	1	PreSIM
Hyde et al. (2022)		Reentry	5 and N/A	4	0 and 5
Yakovchenko et al. (2022)			4 and 5	5	4 and 5
Betancourt et al. (2022)		Substance Use Disorders	5	1	Gen
Blonigen et al. (2020)			5	5	5
Boit et al. (2019)			3 and N/A	Gen	Gen
Brooke and Peck (2019)			4	1	3 and PreSIM
Chang et al. (2018)			Gen	Gen	Gen
Finlay et al. (2022)			1 through 5 and N/A	5	Gen
Rosenheck et al. (2021)			5	PreSIM	PreSIM
Santangelo et al. (2022)			3	Gen	PreSIM
Spence et al. (2020)			5	5	4 and 5
Taylor et al. (2019)			1 through 5 and N/A	Gen	Gen
Timko et al. (2022)			5	5	5
Holliday et al. (2021b)		Suicide	5	5	5
Palframan et al. (2020)			5	1 through 5	Gen
Clary et al. (2020)		Treatment Court	3	PreSIM	3
Head and Woodruff (2020)			3	Gen	3
Derrick et al. (2018)		Veterans Treatment Courts	3	3	3
Douds and Hummer (2019)			3 and N/A	3	3
Morgan et al. (2021)			4	1	1
Smelson et al. (2020)			3	3	3
Tsai et al. (2018)			5	3	3
Finlay et al. (2019b)		Violence	4	1 and PreSIM	4
Lindenfeld et al. (2021)			3	3	3
Paden et al. (2021)			5	Gen	5
Pethrus et al. (2019)			5	Gen	Gen
Schaffer and Zarilla (2018)			4	1 through 5	Gen
Stacer and Solinas-Saunders (2018a)			3	3	4
Elbogen et al. (2022)	Post-deployment health	Multiple Mental Health, Substance Use Disorder and/or Medical Conditions	PreSIM through 5	1 through 5	PreSIM
S. B. Holliday et al. (2022a)	Psychosocial	Multiple Mental Health, Substance Use Disorder and/or Medical Conditions	4	1, 4, and 5	5
Kelton et al. (2022)			5	Gen	PreSIM
Moorhead (2021b)			5	PreSIM	PreSIM
Hyde et al. (2022)		Reentry	4 and 5	4	4
LePage et al. (2018)		Vocational training	5	4	5
LePage et al. (2020)			5	5	5
LePage et al. (2021a)			5	4	5
Blosnich et al. (2020)	Social Determinants of Health	Death	5	Gen	Gen
Lin et al. (2022)		Multiple Mental Health, Substance Use Disorder and/or Medical Conditions	5	4	Gen
Alemi et al. (2020)		Suicide	5	3	Gen
Blosnich et al. (2019)			5	Gen	Gen

Intercept 0 (Community and Emergency Services Intercept), Intercept 1 (Law Enforcement Intercept), Intercept 2 (Initial Detention and Court Hearings Intercept), Intercept 3 (Jails and Courts Intercept), Intercept 4 (Reentry Intercept), Intercept 5 (Community Corrections and Support Intercept), Gen (General), PreSIM (Before involvement with V-SIM), N/A (Not Applicable, Study Sample included Staff Members)

Most studies utilized an observational quantitative design, whereas observational qualitative designs and randomized controlled trials were rare. Of the 89% (95/107) studies with an observational quantitative research design, 14% (13/95) were prospective cohort studies (e.g., Hawks et al., 2020; Miles et al., 2021). Nine studies (8% of 107) used qualitative research methodology and either interviewed participants or utilized focus groups (e.g., Goggin et al., 2018; Morse et al., 2021). Finally, 2% (2/107) of the studies used randomized clinical trial designs (LePage et al., 2020, 2021a) with one additional study (1%) conducting a secondary or sub-group analysis of a randomized clinical trial (LePage et al., 2018).

### Healthcare categories

#### Mental health conditions

Sixty-two percent (66/107) of the included articles covered mental health conditions (Table 1). The articles were dispersed into the following subcategories: 32% (21/66) multiple mental health, substance use disorders and/or medical conditions, 17% (11/66) substance use disorders, 14% (9/66) mental health programming, 9% (6/66) violence, 6% (4/66) post-traumatic stress disorder and/or trauma, 3% (2/66) reentry, 3% (2/66) suicide, 3% (2/66) treatment courts, 8% (5/66) Veterans Treatment Courts, and 6% (4/66) other mental health topics.

Studies of multiple mental health, substance use disorders and medical conditions were distributed by the intercept point of interest at analysis between every intercept except intercept 0 (Community and Emergency Services Intercept) and intercept 1 (Law Enforcement Intercept) (Table 1). The recommendation for intercept interventions was not as varied with almost half of the articles (48%, 10/21) making general recommendations – that is, recommendations that are not specific to any intercept point. For example, Elbogen et al. (2018) collected data from veterans in the community who had a history of incarceration (intercept 5, Community Corrections and Support Intercept) but recommended screening and interventions that were not linked to specific points on the V-SIM model (coded as general). The other half of the articles were split between intercepts 4 (Reentry Intercept), 5 (Community Corrections and Support Intercept), and PreSIM. Articles in this category covered a combination of mental health diagnoses such as post-traumatic stress disorder, traumatic brain injury, and substance use disorder in addition to social and economic stressors, like homelessness or access to care. Articles were split between discussing programming while incarcerated and community reintegration support for veterans with multiple mental health diagnoses (e.g., Barry et al., 2018; MacDonald et al., 2022) and documenting their findings more broadly about understanding the challenges legal-involved veterans with multiple mental health conditions face to improve care (e.g., Bhalla et al., 2020; Chintakrindi & Gupta, 2021; Fletcher et al., 2022). Three articles touched on identifying mental health and behavioral needs to improve transition from military service (PreSIM) (Barr et al., 2022; Edwards et al., 2022c; Ross et al., 2018).

Within the topic of mental health, eleven studies focused on substance use disorders. These articles primarily gathered data from intercept 5 (Community Corrections and Support Intercept), analyzed data from intercept 5 and general analyses, and made recommendations for intercepts 5, PreSIM, and general recommendations (Table 1). Three articles evaluated substance use disorder treatment programs specifically for veterans recently released from prison/jail with the goal of reducing recidivism (Blonigen et al., 2020; Spence et al., 2020; Timko et al., 2022). Four articles studied substance use disorder programs more generally looking at factors that would improve substance use disorder treatment completion, maximize abstinence from substances, motivate veterans for treatment, and enhance care between mental health and substance use disorder treatment (Betancourt et al., 2022; Boit et al., 2019; Chang et al., 2018; Finlay et al., 2022). Finally, three articles focused on substance use disorder problems veterans face post-military (Brooke & Peck, 2019; Rosenheck et al., 2021; Santangelo et al., 2022) and one article evaluated substance use disorder treatment in women legal-involved veterans (Taylor et al., 2019).

Mental health programming was the primary topic of nine articles with a focus on evaluating specific mental health programs, including cognitive-behavioral treatments and psychotherapy programs, such as Moral Reconation Therapy, interventions that enhance social support, service dog training programs, the benefits of veterans’ service units, Residential Rehabilitation Treatment Programs, and dialectical behavior therapy (e.g., Blonigen et al., 2022; Edwards et al., 2022a; Goggin et al., 2018). Findings of these articles varied in the intercepts of the V-SIM for which they had recommendations; however, common among them was that these programs helped veterans prepare for reentry (intercept 4, Reentry Intercept) once they were already in the community (intercept 5, Community Corrections and Support Intercept), and some spoke more generally of the positive impact mental health programs have. The other three articles that studied mental health programming described the importance of identifying veterans, whether upon entry into the criminal legal system (Intercept 0 [Community and Emergency Services Intercept] and intercept 1 [Law Enforcement Intercept]) to allow for early intervention services (Short et al., 2018), or during incarceration and the first weeks of reentry (intercepts 4 [Reentry Intercept] and 5 [Community Corrections and Support Intercept]) (Kim et al., 2019a; Morgan et al., 2019).

#### Access and utilization

Half of the articles (50%, 5/10) studying access and utilization included staff members (VHA staff, VJP specialists, and lead attorneys) as part of their sample in data collection. The intercept point of interest at analysis primarily made general analyses about the services utilized, and seven out of the ten articles made general recommendations (Table 1). Barriers and facilitators of care was the most studied topic in the category of access and utilization and included reentry and the need to increase veteran access to such programs (Kim et al., 2019b; Simmons et al., 2022), the importance of legal clinics and interventions (Timko et al., 2020b, c; Tsai et al., 2018a), and breaking down stigma and enhancing medication knowledge and education to improve medications for opioid use disorder and opioid use disorder treatment (Finlay et al., 2020; Morse et al., 2021; Taylor et al., 2022). Two articles focused on healthcare utilization of minoritized populations, including women and legal-involved veterans, facing difficulties receiving the same quality of substance use disorder treatment than other veterans (Finlay et al., 2021) and veterans dealing with higher healthcare costs than nonveterans (Harris et al., 2021).

#### Psychosocial

Of the seven articles studying psychosocial topics, there was an almost even split, with articles evaluating vocational training and articles studying multiple mental health, substance use disorder and/or medical treatment (Table 1). Unlike the other categories, there was more variation in study designs for this topic: two prospective cohort studies, one qualitative study, one randomized clinical trial, with one secondary or subgroup analysis of a randomized clinical trial, and two other observational designs. The majority used intercept 4 (Reentry Intercept) as the intercept of interest at analyses but the recommendations were rooted in intercept 5 (Community Corrections and Support Intercept). Articles studying vocational training demonstrated the importance of vocational programming to increase employment for formerly incarcerated veterans (LePage et al., 2018, 2020, 2021a). Understanding the various psychosocial factors related to mental health, substance use disorders, and other medical conditions veterans face was the focus of three articles (S. B. Holliday et al., 2022; Kelton et al., 2022; Moorhead, 2021b). Finally, there was an article evaluating the need to understand pre-incarceration life experiences of veterans to better assist them with reentry post-incarceration (PreSIM) (Hyde et al., 2022).

#### Medical

Seven articles looked at medical conditions for legal-involved veterans. Six of the seven used intercept 4 (Reentry Intercept) for the point of data analyses. Of the articles evaluating medical conditions, two focused on improving programming and treatments during incarceration and upon release (Drapela et al., 2019; Hawks et al., 2022), two focused on improving linkage to medical care after their release (Khan et al., 2019; McCall & Tsai, 2018a), one focused on HIV (Hawks et al., 2020), one focused on screening for cognitive dementia (Kuffel et al., 2022), and one study looked at different factors affecting employment for veterans with spinal cord injury (Ottomanelli et al., 2022).

#### Social determinants of health

This category was added to the present scoping review to reflect the growth in interest in social determinants of health. Of the four articles in this category, two noted that adding social determinants of health factors in the electronic health record could help prevent or predict suicide (Alemi et al., 2020; Blosnich et al., 2019). All four articles made general recommendations for intercept intervention. One article used electronic health record data to study death patterns, with the recommendation that prevention efforts be built around social determinants of health (Blosnich et al., 2020). Finally, one study compared social factors between veterans with or without schizophrenia (Lin et al., 2022).

#### Homelessness

Homelessness among veterans with legal criminal involvement was the focus of six studies (Table 1). Two studies looked at general services utilized, and general psychosocial factors associated with homelessness which resulted in general recommendations, without mention of any point of the V-SIM (Harris et al., 2018; Kertesz et al., 2021). Three of the six studies focused on the importance of post-incarceration reintegration, looking at programs provided during incarceration (intercept 4, Reentry Intercept) or programs available post-release from jail/prison (intercept 5, Community Corrections and Support Intercept) (Byrne et al., 2022; LePage et al., 2021b; Szymkowiak et al., 2022). Only one article studying homelessness focused on targeted prevention efforts to reduce the risk of criminal legal involvement and entry into the criminal legal system (PreSIM) (Orak et al., 2022).

### Mapping to the V-SIM

Table 1 gives an overview of all the articles in our review, including their mapping to the V-SIM, and Table 2 tabulates the articles mapped to the V-SIM. Although half of the studies (50%, 54/107) collected data from intercept 5 (Community Corrections and Support Intercept), they analyzed general factors (30%, 32/107) and made general recommendations (36%, 38/107) (Table 2). Only a few articles used the same intercept point for data collection, data analyses, and recommendations. Of these, 5% (5/107) used intercept 3 (Jails and Courts Intercept) for all three categories (e.g., Lindenfeld et al., 2021); 4% (4/107) used intercept 4 (Reentry Intercept) (e.g., Drapela et al., 2019), and 7% (7/107) used intercept 5 (Community Corrections and Support Intercept) (e.g., Byrne et al., 2022) for all three categories. No articles collected data, analyzed data, or recommended interventions for intercept 2 (Initial Detention and Court Hearings Intercept). The N/A option was only used in the “intercept point data were collected from” category, such as a survey for VHA staff who conduct outreach with legal-involved veterans (e.g., Taylor et al., 2022).

**Table 2:** Mapping to the Veterans-Sequential Intercept Model

	**Intercept point data are collected from **	**Intercept of interest at analysis**	**Recommendation for intercept intervention**
N/A	7% (7/107)	0%	0%
Gen	1% (1/107)	30% (32/107)	36% (38/107)
0	0%	0%	1% (1/107)
1	0%	10% (11/107)	3% (3/107)
2	0%	0%	0%
3	8% (9/107)	8% (9/107)	8% (9/107)
4	14% (15/107)	17% (18/107)	12% (13/107)
5	50% (54/107)	14% (15/107)	15% (16/107)
PreSIM	0%	4% (4/107)	9% (10/107)
Multiple	20% (21/107)	17% (18/107)	16% (17/107)

### Intercept point that data were collected from

Of the 107 articles included in this review, 50% (54/107) collected data from intercept 5 (Community Corrections and Support Intercept) when veterans had exited the criminal legal system (e.g., Morse et al., 2021; Timko et al., 2020c), followed by articles that collected data from multiple points (20%, 21/107) (e.g., Kuffel et al., 2022; MacManus et al., 2021), and then studies that collected data from intercept 4 (Reentry Intercept) (14%, 15/107) (e.g., Comartin et al., 2022; Morgan et al., 2021) (Table 1). Articles that collected data from multiple points included various combinations of intercepts; most commonly, intercept 5 and N/A (7%, 7/107), meaning, the study sample included veterans in the community (post-release) and staff members (e.g., Blonigen et al., 2022; Hyde et al., 2022). Six of these twenty-one articles covering multiple intercepts collected data from intercepts 4 (Reentry Intercept) and 5 (Community Corrections and Support Intercept), meaning incarcerated veterans and veterans in the community post-release were included in the sample (e.g., Logan et al., 2021a, b; MacDonald et al., 2022). Seven studies (7%) did not collect data by any intercepts and were coded as N/A since the sample included VHA staff, staff members of a reentry organization, or lead attorneys (e.g., Blonigen et al., 2021; Weaver et al., 2022). No articles gathered data from intercepts 0 (Community and Emergency Services Intercept), 1 (Law Enforcement Intercept), or 2 (Initial Detention and Court Hearings Intercept). Only two articles (2%) collected data from different timepoints or prospectively followed veterans from before the V-SIM (PreSIM) through intercept 5 (Community Corrections and Support Intercept) (Elbogen et al., 2022; MacManus et al., 2021).

### Intercept of interest at analyses

The majority of articles (30%, 32/107) conducted general analyses, meaning they evaluated general factors, treatments, or services without linking to one intercept point (e.g., Blosnich et al., 2019; Davis et al., 2020; Paden et al., 2021) (Table 1). In addition, 17% (18/107) of articles focused on intercept 4 (Reentry Intercept) for data analysis (e.g., Khan et al., 2019; Sylvia et al., 2021) and 17% (18/107) on multiple intercept points at analysis (e.g., Camins et al., 2022; Elbogen et al., 2022). Of the eighteen articles covering multiple intercept points, six (6%) analyzed coordination challenges of reentry and community reintegration support (intercepts 4 and 5) (e.g., Simmons et al., 2022). Eight articles covered the whole V-SIM process, with six analyzing data from intercepts 1 (Law Enforcement Intercept) through 5 (Community Corrections and Support Intercept) (e.g., Schaffer & Fulmer, 2018; Taylor et al., 2022) and two focusing on intercepts 2 (Initial Detention and Court Hearings Intercept) through 5 (Community Corrections and Support Intercept) (MacDonald et al., 2022b; Rodriguez et al., 2019). Five articles analyzed data from before veterans entered the V-SIM (e.g., Moorhead, 2021b), one of these comparing PreSIM data to intercept 1 (Law Enforcement Intercept) (Finlay et al., 2019b). No data were collected during the PreSIM period; however, four articles included PreSIM as the intercept of interest at analysis. Finally, no articles analyzed data from intercept 0 (Community and Emergency Services Intercept) or 2 (Initial Detention and Court Hearings Intercept).

### Recommendation for intercept intervention

Like the intercept of interest at analysis, most studies (36%, 38/107) made general recommendations for the intercept intervention (e.g., Schaffer & Zarilla, 2018; Wang et al., 2019). This was followed by 16% (17/107) of articles that made recommendations for multiple intercept points (e.g., MacDonald et al., 2022; Rodriguez et al., 2019) and 15% (16/107) that made recommendations for intercept 5 (Community Corrections and Support Intercept) (e.g., Blonigen et al., 2021; Holliday et al., 2021b). Of the articles providing recommendations for multiple intercepts, eleven had recommendations for intercepts 4 (Reentry Intercept) and 5 (Community Corrections and Support Intercept) (e.g., Kim et al., 2019a; Spence et al., 2020). Nine articles (8%) provided recommendations for intervention at intercept 3 (Jails and Courts Intercept) of the V-SIM. Of these, seven out of nine focused specifically on Veterans Treatment Courts or treatment courts (e.g., Douds & Hummer, 2019; Head & Woodruff, 2020). Ten articles (9%) made recommendations for PreSIM, meaning their conclusions were catered towards community reintegration after military service or the military-to-civilian transition to prevent entrance into the criminal legal system (e.g., Miles et al., 2021; Santangelo et al., 2022).

## Discussion

This scoping review provides an update summarizing the research literature on legal-involved veterans’ health and healthcare from December 2017 to December 2022. As in the prior scoping review, articles primarily focused on mental health conditions and treatment and used an observational quantitative study design. The V-SIM was developed to be used by various key partners to identify places where they can intervene with legal-involved veterans and to determine gaps in services. When mapping to the V-SIM, it became evident that most of the recent research used intercept 5 (Community Corrections and Support Intercept) to gather data but made analyses and recommendations for other intercept points or made general recommendations not specific to an intercept point. There were no studies focused on early intercept points of 0 (Community and Emergency Services Intercept), 1 (Law Enforcement Intercept), and 2 (Initial Detention and Court Hearings Intercept); therefore, there is limited scientific evidence to guide prevention and early intervention efforts offered at these intercept points.

### Comparison to original scoping review and gap assessment

The first scoping review published in 2019 identified 191 articles over a 70-year period (1947 to 2017). In this study, we identified 107 articles published within the last five years (2018 to 2022). Over 60% of the articles reviewed, studied veterans’ mental health conditions, 92% of studies (limited to reports in English) were conducted in the United States, and 89% used an observational quantitative research design (Appendix 1). Some gaps identified in the prior study were partially addressed in the last five years. For example, there were six new articles published on healthcare organization and delivery, compared to two articles from the prior review. There was also an increase in the number of studies that conducted separate analyses by sex and included details specifically for women or only included women in their sample (Blosnich et al., 2020; Brooke & Peck, 2019; Holliday et al., 2021b; McCall & Tsai, 2018a; Stefanovics et al., 2022; Taylor et al., 2019).

Sociodemographic differences, however, are still not a priority in the field. Limited research evaluated rural veterans (e.g., Finlay et al., 2018b), older veterans (e.g., Barry et al., 2018), or veterans of color (e.g., Browne & Mohamed, 2021). Only one article studied transgender veterans (Fletcher et al., 2022). Without further research on different demographic groups of veterans involved in the legal system and their experiences in correctional and other criminal legal settings, it will be difficult to develop programmatic efforts to address their treatment and housing needs. Related to sociodemographic differences, we added a new healthcare topic of social determinants of health, showcasing the growth in this field. While there was an increase of articles researching various social determinants of health (four articles compared to zero), more work is needed in this area to fully understand it.

### Gaps by V-SIM intercept point

There is a variety of disciplines that have an interest in legal-involved veterans, including the VHA and community healthcare providers, criminal justice staff across a variety of settings (e.g., law enforcement, courts, and correctional settings), and veterans themselves. We used the V-SIM as our conceptual model because front-line practitioners and clinical leaders use this model to guide their outreach activities and resource deployment. Furthermore, the Justice-Involved Veterans Network spent years developing consensus across key partners in creating the V-SIM model. We attempted to map all studies in this scoping review to the V-SIM intercept points but ultimately, found many gaps in the research.

#### Intercepts 0 and 1

Although conceptualized as separate intercepts, many deflection programs include elements of intercepts 0 (Community and Emergency Services Intercept) and 1 (Law Enforcement Intercept); therefore, we grouped them here together. Starting in Fiscal Year 2023, the VHA began a series of trainings focused on community law enforcement-based deflection (Department of Veterans Affairs, 2023). Interdisciplinary teams of VJO staff, mental health treatment staff, and VA law enforcement officers were trained in deflection practices with the goal of developing deflection programming at their local VA in collaboration with local community partners.

Research on veteran deflection programs is scant, reflecting that this is a relatively new area of work. In 2023, the VHA conducted a brief survey of VJO staff to capture current veteran deflection activities. The vast majority (96%) of respondents reported that interactions with law enforcement were on an as-needed basis even though 43% had received Crisis Intervention Training (Washington, Singh, Stewart, Firesheets, Charlier, & Finlay: Veterans deflection: not waiting for military veterans to be arrested or in crisis before we act, forthcoming). In a study of two veteran-specific deflection programs, veterans used more healthcare services six months after deflection compared to before; however, there was no comparison group of veterans who did not receive deflection, so program effectiveness could not be estimated (Tsai et al., 2023). Randomized controlled trials and high-quality observational studies will be needed to answer which deflection programs will be effective for veterans who encounter crisis services and law enforcement in intercepts 0 and 1. Community deflection programs also lack evaluation with only 17% of opioid response deflection initiatives in the United States reporting formal evaluations (Ross & Taylor, 2022). Crisis Intervention Teams, which are a specific form of deflection programming that bring law enforcement and mental health providers together in community collaborations, have had no effect on arrest rates (Taheri, 2016), suggesting that community-based research on this topic will not provide much guidance on effective programming.

#### Intercept 2

We did not identify any articles in intercept 2 (Initial Detention and Court Hearing Intercept). There are opportunities for diversion away from the legal system after entering it, such as screening for mental health and substance use disorder diagnoses, and connection to community services (e.g., housing) (Johnson, 2022). There may already be screening and programming happening in criminal legal agencies at this time point, but without a comprehensive assessment and evaluation of these programs, it is not possible to recommend any for further expansion and adoption.

#### Intercept 3

Veterans Treatment Courts, a type of problem-solving court, are among the most examined intervention for legal-involved veterans in intercept 3 (Jails and Court Intercept). However, much of those studies focus on legal measures without including healthcare and were excluded from our study. Furthermore, there has never been a randomized controlled trial of Veterans Treatment Courts and the courts vary widely in the veterans they serve and the services they offer (McCall et al., 2018; Timko, 2017).

Pretrial detainment in jails is also part of intercept 3, but there are no studies we are aware of that focus on veterans in jails pretrial. There are over 3,000 jails across the country, and like Veterans Treatment Courts, they vary widely in programs offered. Without any information on veteran-specific programming in pretrial detention settings, it is challenging to offer recommendations for future research other than to start with an initial survey to understand what is happening.

#### Intercept 4

Intercept 4 (Reentry Intercept) has historically focused on reentry services, but we expanded this intercept to include programs that occur during incarceration in correctional settings. Although the VHA is prohibited from providing healthcare services to veterans while they are under the care of another agency, veteran-specific programming offered by correctional agencies and local community organizations have proliferated. For example, Veteran Dorms (also called Pods or Hubs) have been created in prisons and jails to house veterans together and more efficiently and effectively deliver services (Benos, 2019; Tsai & Goggin, 2017). Research in this area is at the beginning stages and there are no rigorous evaluations or randomized controlled trials that we are aware of. Similarly, reentry services and programs exist for veterans exiting incarceration, such as the Veterans Justice Programs, but there have been no evaluations of these programs that would help guide future efforts to support veterans during the reentry process.

#### Intercept 5

More than 50% of the articles included in this scoping review gathered data from intercept 5 (Community Corrections and Support Intercept) but made conclusions for different intercepts. Much of the research was conducted with veterans in healthcare settings asking them to report on their history of legal involvement. More prospective studies and studies evaluating existing treatment programs are needed to answer questions about what services veterans need while under community supervision and after they are no longer formally involved with the legal system.

#### General

We utilized the term general to indicate articles that conducted studies that were too vague in the intercept point they assessed or that could not clearly be matched to an intercept point. While this approach of having general data analyses and general recommendations is still informative to provide a broad overview for legal-involved veterans, without the details at the intercept points, it becomes difficult to guide future programming targeted at the specific points of the V-SIM. One critique of the SIM is that legal system involvement is not linear, though the model is depicted as linear. Perhaps this assessment partially explains why much of the research lacks specificity of intercept points.

#### Multiple

Multiple articles in the present review focused on providing general recommendations without tying to a specific intercept point. Oftentimes, data were collected, analyzed, and recommendations were provided for multiple intercepts of the model, often blurring from one intercept to the other. There were multiple instances where we were unclear on how to code which intercept the article was focused on as intercepts can be subjective, straddle multiple intercept points, or span across the V-SIM. For example, Brooke and Peck (2019) used data from incarcerated veterans (intercept 4, Reentry Intercept) but made recommendations for gender-specific programming during the transition from military to civilian life (PreSIM) and for Veterans Treatment Courts (intercept 3, Jails and Courts Intercept).

#### PreSIM

PreSIM was used to denote studies where the recommendations were to provide healthcare during the period after military discharge and prior criminal legal involvement to prevent any legal contact. Many articles suggested the need to identify veterans sooner in the criminal legal system continuum so they can receive treatments and resources earlier. It is not simple to identify veterans earlier in the V-SIM. A lack of a clear definition for ‘veteran’ coupled with reliance on self-identification from veterans makes it difficult to provide resources at earlier points in the criminal legal system, such as when veterans are interacting with law enforcement or other crisis services (Seamone, 2023). The Veterans Justice Commission, launched in 2022 with the purpose of recommending evidence-based policy changes for veterans involved in the legal system (*Council on Criminal Justice*, 2023a). Their report highlighted the need to identify veterans as early as possible in their criminal legal involvement, such as having law enforcement utilize veteran specific search services (Council on Criminal Council on Criminal Justice, 2023b).

### Uptake of the V-SIM

Conceptual models are used within research to guide organization and design of a study and ground findings in the existing literature (Mock V., 2007). In the area of legal-involved veterans, conceptual models are absent from most research studies. However, the V-SIM is used by other sectors that have an interest in veterans involved in the criminal legal system, including the legal system itself (where SIM concepts are well known) and the healthcare/treatment system (where SIM concepts are becoming better known). We recommend that researchers, along with other practitioners working in this space, adopt the V-SIM as a unifying conceptual model. There are multiple benefits from adopting a single model: (1) ease of communication with shared concepts and terminology, (2) data collection and measurement focused on the same variables and outcomes, (3) explicit identification of research and knowledge gaps, (4) generation of results that fill identified gaps and inform programs, and (5) resource development. Currently, there were few studies that explicitly identified the V-SIM intercept point they were studying and almost no studies testing programs at those intercept points. Without this information, practitioners have no guidance on evidence-based practices they should implement. Efforts are needed to disseminate and encourage researchers and other key partners to adopt the V-SIM in their work. The research area of dissemination and implementation science may provide some ideas for how to increase update of this conceptual model, including models such as the Diffusion of Innovation, and the Conceptual Framework for Research Knowledge Transfer and Utilization (Tabak et al., 2012). However, it is challenging to increase adoption of anything, whether it is a conceptual model or evidence-based program, and strategic efforts will be needed across key partners to ensure use of the V-SIM.

### Limitations

There are a few limitations to this scoping review. First, the search engines were limited to healthcare databases such that there may be other articles in sociology, law, and criminal legal search engines relevant to legal-involved veterans that we did not identify. Second, only English publications were included in our study, limiting the generalizability of findings to non-English speaking settings. While we included non-US studies that were published in English, unclear is the extent to which the V-SIM can be used outside the United States. Different countries may have different criminal legal systems and services available for legal-involved veterans. For example, Pethrus et al. (2019) evaluated violent crime convictions in Sweden among veterans and MacManus et al. (2021) evaluated criminal offending among veterans in the United Kingdom. Any conclusions drawn from those studies may not be relevant to veterans in the United States legal system. It is beyond the scope of our study to investigate other countries’ legal systems. Finally, we also included articles about civil legal clinics as they work with legal-involved veteran populations. However, civil and criminal legal issues are different, though some veterans who sought help in civil legal clinics needed help with criminal issues (Timko et al., 2020b, c). We included these articles in the scoping review, but more work is needed to tease apart civil and criminal legal issues among veterans.

## Conclusion

Developing an updated resource of literature on the health and healthcare of legal-involved veterans is essential to inform current research, discover gaps, and highlight areas for future research. While some gaps in research were filled since our last review, such as an increase of studies focused on women veterans and on healthcare organization and delivery, other gaps remain, such as limited work on other sociodemographic groups (e.g., rural veterans, older veterans). Randomized controlled trials and observational quantitative studies with comparison groups are still very rare in this area. To recommend evidence-based programs, these kinds of studies are needed. Finally, researchers need to clarify what intercept they are collecting and analyzing data from to make specific recommendations. The V-SIM is widely used by front-line practitioners and should be used more effectively to guide research.

### Supplementary Information

**Supplementary Material 1.**

**Supplementary Material 2.**

## Data Availability

Table 1 cites all articles included in this scoping review which are published in peer-reviewed journals.

## Abbreviations

HCRV: Health Care for Reentry Veterans
SIM: Sequential Intercept Model
V-SIM: Veterans-Sequential Intercept Model
VA: U.S. Department of Veterans Affairs
VHA: Veterans Health Administration
VJO: Veterans Justice Outreach
VJP: Veterans Justice Programs
