# Supplementary material for: Scoping review of military veterans involved in the criminal legal system and their health and healthcare: 5-year update and map to the Veterans-Sequential Intercept Model
Source: Health Justice. 2024 Apr 19;12:18. doi: 10.1186/s40352-024-00274-9 (PMC11027330; doi:10.1186/s40352-024-00274-9)
Supplement: Supplementary file 2 — Supplementary Material 2. [file 40352_2024_274_MOESM2_ESM.docx]

**Appendix 1: Comparison Between Original Review and Update**

|  | **1947 – 2017 Articles** | **2018 – 2022 Articles** |
| --- | --- | --- |
| Comparison of Study Design |  |  |
| Observational Design | 91% (173/191) | 89% (95/107) |
| Randomized Clinical Trial Design | 5% (10/191) | 2% (2/107) |
| Secondary Analysis of Randomized Clinical Trial | 1% (2/191) | 1% (1/107) |
| Qualitative Interviewing | 3% (6/191) | 8% (9/107) |
| Comparison of Sample Size |  |  |
| <100 participants | 21% (41/191) | 26% (28/107) |
| 100-1000 participants | 40% (77/191) | 23% (25/107) |
| >1000 participants | 38% (73/191) | 50% (54/107) |
| Comparison of Healthcare Categories |  |  |
| Mental Health | 68% (130/191) | 62% (66/107) |
| Homelessness | 13% (24/191) | 6% (6/107) |
| Medical | 5% (10/191) | 7% (7/107) |
| Access and Utilization | 7% (14/191) | 9% (10/107) |
| Psychosocial | 5% (10/191) | 7% (7/107) |
| Healthcare Organization & Delivery | 1% (2/191) | 6% (6/107) |
| Long Term Care/ Aging | 1% (1/191) | 0% |
| Post Deployment Health | 0% | 1% (1/107) |
| Social Determinants of Health | 0% | 4% (4/107) |

**Appendix 2: Data Extraction Study Characteristics**

| ***Healthcare Category***  Subcategory |
| --- |
| Healthcare Category |
| ***Mental Health***  Homelessness  Mental healthcare programming  Multiple mental health, substance use disorder and/or medical conditions  Other mental health topics  PTSD and/or trauma  Race/ethnicity  Substance use disorders  Suicide  Treatment Courts  Veterans Treatment Courts  Violence  ***Homelessness***  Death  Multiple mental health, substance use disorders and/or medical conditions  Other mental health topics  PTSD and/or trauma  Substance use disorders  Violence  Vocational training  ***Access and Utilization***  Barriers and facilitators to care  Death  Health care utilization  Veterans Treatment Courts  Violence  ***Medical***  Brain Injury  Death  Hypertension  Infectious diseases  Other medical topics  ***Psychosocial***  Homelessness  Multiple mental health, substance use disorders and/or medical conditions  PTSD and/or trauma  Screening in primary care  Vocational training  Reentry  ***Healthcare Organization & Delivery***  Mental healthcare programming  Peer support  Electronic Health Record  ***Long-term Care/Aging***  Mental health programming  ***Post Deployment Health***  Healthcare programming |
| Study Design |
| Randomized Controlled Trial  Secondary or sub-group analysis of Randomized Controlled Trial  Observational (prospective cohort)  Observations (Other) |
| Sample Size |
| N < 100  N = 100 – 1,000  N > 1,000  N/A |
| % Veterans |
| ≤ 10%  11 – 50% 51 – 99%  100%  N/A |
| % Legal-Involved Veterans |
| ≤ 10%  11 – 50% 51 – 99%  100%  N/A |
| Reported Sex |
| Yes  No |
| Reported Race |
| Yes  No |
| Reported Age |
| Yes  No |
| Research Setting |
| Single site VA Multi-site VA Administrative database VA Single site court  Multi-site court  Prison  Jail  Non-VA healthcare setting  Multiple |
| Period of Military Service |
| OEF/OIF/OND/Persian Gulf  Vietnam  WWII Not specified  Multiple periods included |
| Population |
| Veterans  Clinics  Providers  Policy  Criminal Justice staff  Other |
| Outcomes Reported |
| Clinical  Resource utilization  Criminal justice  Cost  Other |
| Funding Source |
| Department of Veterans Affairs (VA) Department of Defense (DOD) National Institute of Health (NIH) National Institute of Justice/Bureau of Justice Assistance (NIJ/BJA)  Other government  Foundation  Industry  University  Not reported  Unfunded |
| Country |
| USA Canada  United Kingdom  Australia  Other (write in) |
| Period of Data Collection |
| Write in |

Note. Department of Veterans Affairs (VA), Post-Traumatic Stress Disorder (PTSD), Operation Enduring Freedom (OEF), Operation Iraqi Freedom (OIF), and Operation New Dawn (OND).
